# A novel transurethral resection technique for superficial bladder tumor: retrograde en bloc resection

**DOI:** 10.1186/s12957-017-1192-6

**Published:** 2017-07-06

**Authors:** Kai-Yan Zhang, Jin-Chun Xing, Wei Li, Zhun Wu, Bin Chen, Dong-Yu Bai

**Affiliations:** 1 Department of Urology, Xiamen University Affiliated First Hospital, 55 Zhenhai Road, Siming District, Xiamen, 361003 China; 2 Department of Pathology, Xiamen University Affiliated First Hospital, 55 Zhenhai Road, Siming District, Xiamen, 361003 China

**Keywords:** En-bloc resection, Non-muscle-invasive bladder cancer, Transurethral resection of bladder tumor

## Abstract

**Background:**

Transurethral resection of bladder tumor (TURBT) is the standard approach to bladder tumors but suffers from several disadvantages. The aim of this study was to evaluate the safety and efficacy of a novel procedure of retrograde en bloc resection of bladder tumor (RERBT) with conventional monopolar resection electrode for the treatment of superficial bladder tumors.

**Methods:**

RERBT and conventional TURBT (C-TURBT) were conducted, respectively, in 40 and 50 patients diagnosed with superficial papillary bladder tumors. In the RERBT group, the tumors were en bloc removed retrogradely under direct vision using a conventional monopolar electrode. Patients’ clinicopathological, intraoperative, and postoperative data were compared retrospectively between the RERBT and C-TURBT groups.

**Results:**

Of the 90 patients, 40 underwent RERBT and 50 underwent C-TURBT. Both groups were comparable in clinicopathological characteristic. RERBT could be performed as safely and effectively as C-TURBT. There were no significant differences in operative time and surgical complications. The cumulative recurrence rates between groups were similar during up to 18 months follow-up. The detrusor muscle could be identified pathologically in 100% of RERBT tumor specimens and the biopsy of tumor bases, but only in 54 and 70%, respectively, of C-TURBT samples (*P* < 0.01).

**Conclusions:**

The RERBT technique is feasible and safe for superficial bladder tumors using conventional monopolar resection setting, with the advantages of adequate tumor resection and the ability to collect good quality tumor specimens for pathological diagnosis and staging compared to conventional TURBT.

**Electronic supplementary material:**

The online version of this article (doi:10.1186/s12957-017-1192-6) contains supplementary material, which is available to authorized users.

## Background

Bladder cancer is the most common and second most common urological cancer in China and Western countries [1], respectively. Seventy percent of bladder cancer are non-muscle invasive at diagnosis [2] and treated by transurethral resection (TUR) of the tumors. Adequate initial resection coupled with accurate histological diagnosis of the resected tumors are essential to successful management of these tumors [3]. To date, transurethral resection of bladder tumor (TURBT) remains the gold standard for the treatment of non-muscle-invasive bladder cancer (NMIBC).

The goal of TURBT is to remove all visible lesions and provide viable tissues for accurate pathological diagnosis [4]. The detection of the detrusor muscle within the tissue was the most important parameter that is associated with recurrence-free survival [5]. However, staging on TURBT specimens is often inaccurate due to their poor quality [6] resulted from piecemeal resection of tumors and charring of the resected tissues by conventional TURBT (C-TURBT) technique [3].

Here we introduce a novel retrograde en bloc TURBT (RERBT) technique using conventional monopolar electrode and compared its safety and efficacy with C-TURBT. To the best of our knowledge, this is the first report on en bloc resection of bladder tumor without any adjustment of electrode loop and use of alternative energy or facilities.

## Methods

### Study population

From October 2014 to December 2015, data of 90 consecutive patients with pathologically diagnosed primary NMIBC after RERBT or C-TURBT in our hospital was reviewed retrospectively. RERBT or C-TURBT was performed by two urologists who were well trained and experienced with endoscopic practices. The cases were divided into two groups: the RERBT group (*n* = 40) and the C-TURBT group (*n* = 50) (Table 1). This study was approved by the Ethics Committee of Xiamen University Affiliated First Hospital.

**Table 1:** Clinicopathological characteristics of patients

Variable		RERBT (*n* = 40)	C-TURBT (*n* = 50)	*P* value
Age (year)		60.65 ± 13.08	60.80 ± 14.04	0.959
Size				0.650
	≤3 cm (*n*)	32 (80%)	38 (76%)	
	>3 cm (*n*)	8 (20%)	12 (24%)	
Gender				0.166
	Male	35 (87.5%)	38 (76%)	
	Female	5 (12.5%)	12 (24%)	
Tumor multiplicity				0.705
	Single	29 (72.5%)	38 (76%)	
	Multiple	11 (27.5%)	12 (24%)	
Tumor morphology				0.686
	Pedunculate	28 (70%)	33 (66%)	
	Flat	12 (30%)	17 (34%)	
Stage				0.119
	Ta	15 (37.5%)	27 (54%)	
	T1	25 (62.5%)	23 (46%)	
Grade (WHO2004)				0.250
	LMP	9 (22.5%)	12 (24%)	
	LG	22 (55%)	23 (46%)	
	HG	9 (22.5%)	15 (30%)	
Location				0.753
	Lateral wall	22 (55%)	22 (44%)	
	Posterior wall	5 (12.5%)	8 (16%)	
	Anterior wall	6 (15%)	7 (14%)	
	Dome	0 (0)	3 (6%)	
	Trigone/bladder neck	4 (10%)	4 (8%)	
	Multiple	3 (7.5%)	6 (12%)	

*RERBT* retrograde en bloc resection of bladder tumor, *C-TURBT* conventional transurethral resection of bladder tumor, *LMP* papillary urothelial neoplasia of low malignant potential, *LG* low-grade papillary urothelial carcinoma, *HG* high-grade papillary urothelial carcinoma

### Surgical procedure

Both RERBT and C-TURBT were performed using the same Circom 25.6F continuous flow resectoscope with monopolar electrode loop (Richard Wolf GmbH, Knittlingen, Germany) and with cutting and coagulation power set at 110 and 75 W, respectively (Valley Lab, USA). All surgeries were performed in lithotomic position under general anesthesia. Tumor resections were performed routinely with a semi-filled bladder (filled with 200–300 mL irrigation fluid). Tumors less than 2.0 cm in diameter were retrieved under siphon effect. For those larger than 2.0 cm, Elik’s evacuator was used to retrieve the specimen. Biopsy of tumor bases and surrounding mucosa was performed routinely with cold cup forceps after the tumors were resected. The resected tumor was submitted for pathologic evaluation. We did not perform a second-look transurethral resection as a routine. The novel RERBT surgical procedure was approved by the Ethics Committee of Xiamen University Affiliated First Hospital.

#### RERBT

**Fig. 1:**
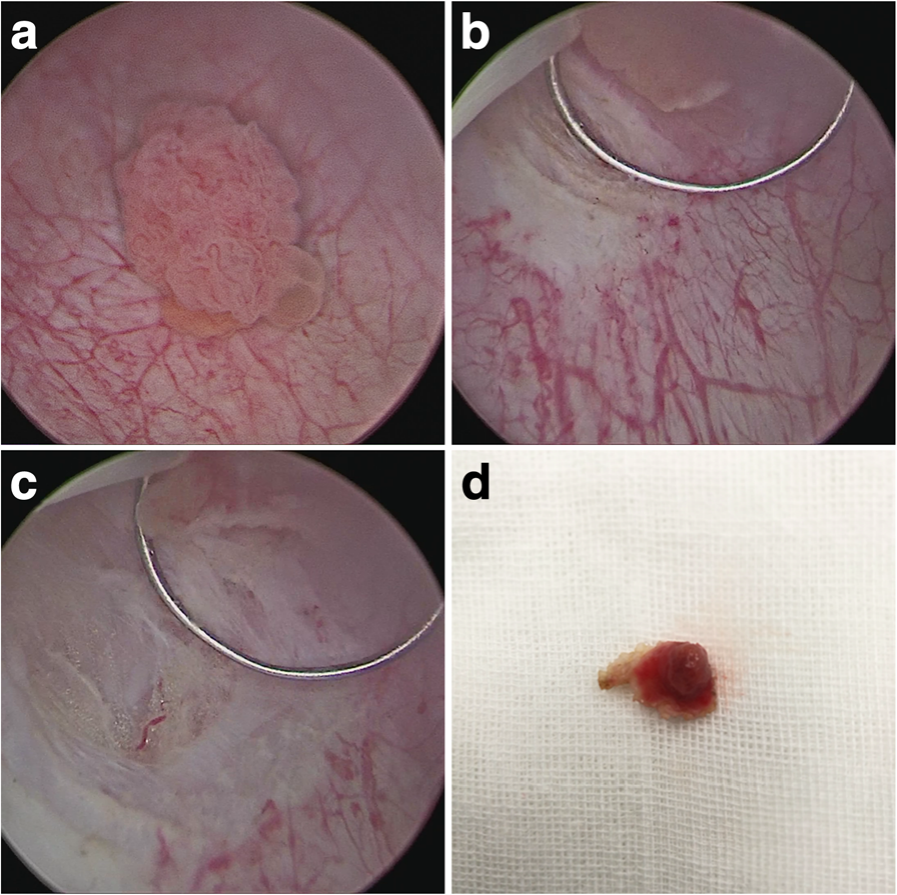
**a** A 1.5-cm-diameter bladder tumor on the right bladder wall. **b** Macroscopic normal mucosa about 0.5 cm away from the tumor base was margined. Then, the bladder mucosa was subsequently cut in a “flash-firing” fashion. **c** After the deep muscle layer was reached when normal glistening yellow fat is seen between muscle layers, the loop was moved forward along the muscle layer. **d** The tumor was resected in one piece


Macroscopic normal mucosa about 0.5–1.0 cm away from the tumor base was margined by using coagulation current. Blood vessels entering the tumor were blocked before resection to reduce intraoperative hemorrhage. Conventional monopolar electrode loop was used to push the bladder wall. After applying gentle pressure, the bladder mucosa was cut in a “flash-firing” fashion, which we called a “small bite.” When the deep muscle layer was reached, usually after one or two “small bites,” the loop was moved forward along the muscle layer using the same “small bite” cutting fashion, the base of the tumor was then lifted and pushed away step by step, and the tumor was resected in one piece retrogradely. All the procedures were performed under direct vision without the tumor body obstructing the view of incision position. The incision depth can be controlled by the amount of pressure applied to the bladder wall and the time of cutting. During cutting, bleeding vessels were coagulated simultaneously, keeping a clear vision of the field (Fig. 1 and Additional file 1).

Additional file 1:
**Video S1.** A patient with a 1.0-cm-diameter lesion on the left bladder wall underwent RERBT. Initially, macroscopic normal mucosa about 0.5 cm away from the tumor base was margined by using coagulation current. Blood vessels entering the tumor were blocked before resection to reduce intraoperative hemorrhage. Conventional monopolar electrode loop was used to push the bladder wall. After applying gentle pressure, the bladder mucosa was cut in a “flash-firing” fashion, which we called a “small bite.” When the deep muscle layer was reached, usually after one or two “small bites,” the loop was moved forward along the muscle layer using the same “small bite” cutting technique, the base of the tumor was then lifted and pushed away step by step, and the tumor was resected in one piece retrogradely. (AVI 34285 kb)


For larger tumors, the protruding tumors were first removed superficially by conventional TURBT, and the remaining stalk and base lesion was removed en block by the novel procedure, avoiding disruption of the tumor base architecture before en bloc or divisional resection by RERBT.

#### C-TURBT

For conventional TURBT, a piece-by-piece resection to the muscle layer was performed.

### Postoperative management

Intravesical instillation was performed with 40 mg pirarubicin dissolved into 50 mL of 5% glucose solution weekly for 8 weeks starting 1 week postoperatively, followed by monthly maintenance to 1 year. Follow-up included ultrasonography, cystoscopic examination, and urinalysis every 3 months for the first year and 6 months thereafter. The histological grades and tumor stages were assigned according to the WHO 2004 classification [7].

### Statistical analysis

Statistical analyses were carried out with IBM SPSS Statistics ver. 20.0 (IBM Co., Armonk, NY, USA). Measurement data was analyzed with Student’s *t* tests. Categorical variables were compared using Pearson chi-square followed by Fisher’s exact test, and for continuous variables, the non-parametric Mann–Whitney test was used. A *P* value <0.05 was considered statistical significant.

## Results

**Fig. 2:**
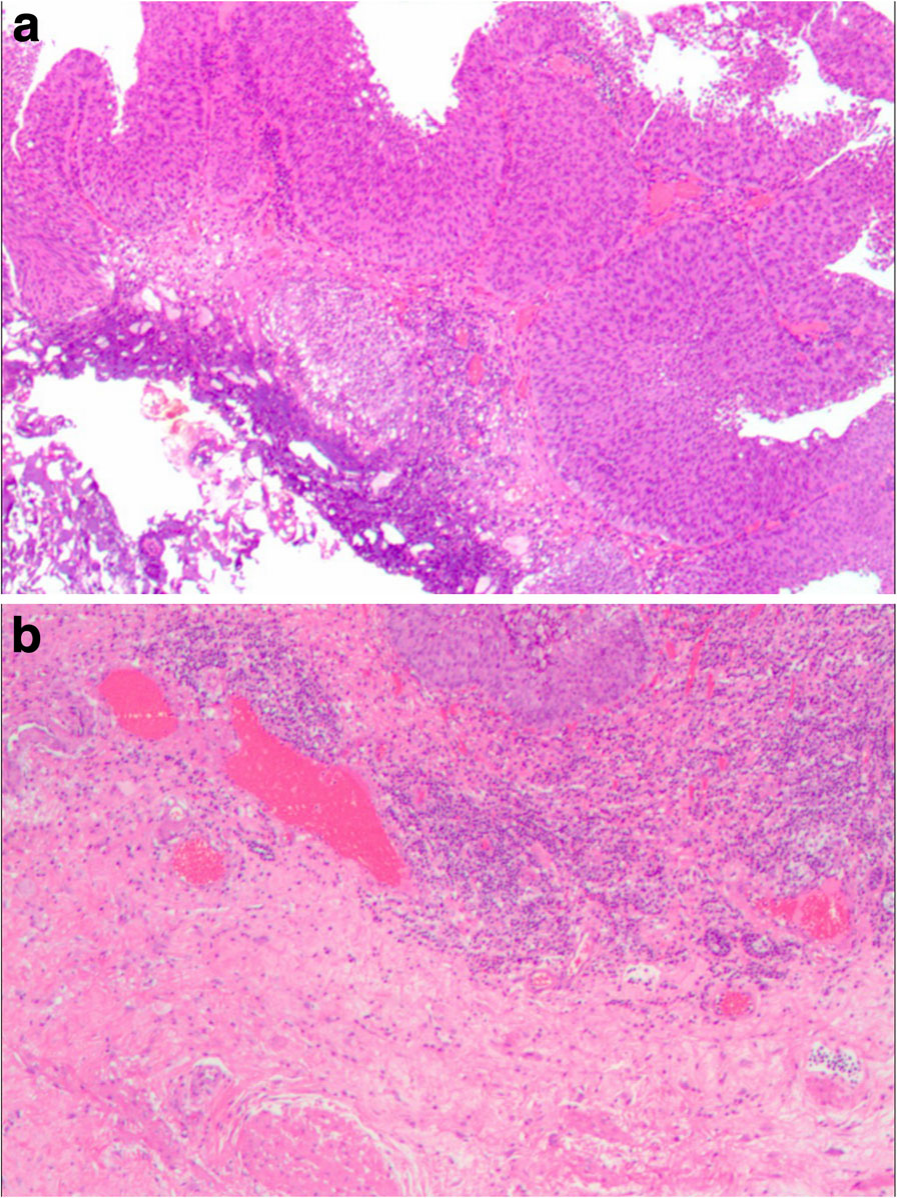
Histologic findings of resected tumors. **a** The lamina propria mucosa was severely charred without muscular propria in a tumor in the C-TURBT group (×40). **b** The lamina propria mucosae remained intact, and the muscular propria was identified in a tumor in the RERBT group (×40)

Forty and 50 patients received RERBT and C-TURBT, respectively. All tumors in both groups were papillary. Blood loss during the procedures was minimal, and no blood transfusion was required in all cases. The two groups had comparable clinicopathological characteristics including gender, age, tumor grade, tumor multiplicity, tumor size, and tumor location (Table 1). Table 2 lists the intra- and postoperative characteristics of RERBT vs. C-TURBT. According to the Clavien–Dindo classification for surgical complications [8], only Grade I and Grade II complications occurred in each group (Table 2). Intraoperative obturator nerve reflex occurred in both groups (22.5 and 24% in RERBT and C-TURBT groups, respectively, *P* = 0.867). Two (5%) patients in the RERBT group developed small bladder perforation which was managed by catheterization for 6 days before discharge. In contrast, four (8%) patients in the C-TURBT group had this complication (Table 2, *P* > 0.05). Pathological examination showed that the detrusor muscle was identifiable in both the tumor specimens and tumor base biopsies of all RERBT patients, suggesting adequate resection of the tumor. However, the detrusor muscle could only be seen in 54% of the tumor specimens and 70% of the tumor base biopsies of C-TURBT patients (*P* < 0.01). In addition, excised tumors showed that the lamina propria in the RERBT group remained intact compared to being severely charred in the C-TURBT group (Fig. 2).

**Table 2:** Perioperative and follow-up data

Variable	RERBT (*n* = 40)	C-TURBT (*n* = 50)	*P* value
Operative time (min)	36 ± 11.8	34 ± 13.6	0.464
Complications			0.7633
Grade I	1 (2.5%)	2 (4%)	
Grade II	6 (15%)	8 (16%)	
Grade III	0	0	
Obturator nerve reflex	9 (22%)	12 (24%)	0.867
Bladder perforation	2 (5%)	4 (8%)	0.689
Presence of the detrusor muscle			
Tumor specimen	(40/40) 100%	(27/50) 54%	0.000
Tumor base	(40/40) 100%	(35/50) 70%	0.000
Irrigation (day)	1.16 ± 0.41	1.22 ± 0.45	0.518
Catheterization (day)	4.25 ± 2.04	4.65 ± 2.16	0.373
Postoperative hospital stay (day)	4.07 ± 0.57	4.18 ± 0.59	0.400
Residual tumor on the base	0	2 (5%)	0.500
Follow-up (months)	10.8 ± 3.9	11.3 ± 4.22	0.775
Cumulative recurrence			
In field	2 (5%)	5 (10%)	0.455
Out of field	6 (15%)	7 (14%)	0.893
Over all	8 (20%)	12 (24%)	0.650
LMP	0	0	-
LG	1 (2.5%)	2 (4%)	0.693
HG	7 (17.5%)	10 (20%)	0.763

*RERBT* retrograde en bloc resection of bladder tumor, *C-TURBT* conventional transurethral resection of bladder tumor, *LMP* papillary urothelial neoplasia of low malignant potential, *LG* low-grade papillary urothelial carcinoma, *HG* high-grade papillary urothelial carcinoma

All cases were performed with at least one episode of the cystoscope during up to 18 months follow-up (3~18 months). The overall cumulative recurrence of RERBT and C-TURBT was 20 and 24%, respectively (*P* = 0.650) (Table 2). Most recurrence occurred in high-grade patients and was likely out of the previous resection field.

## Discussion

TURBT is the standard surgical procedure for non-muscle-invasive bladder cancer [5]. However, it does not follow the basic oncologic surgical principle of en bloc resection with up to 51% of specimens lacking the detrusor muscle and subsequently resulting in a high rate of incomplete resection up to 78% and high recurrence rates (50~70%) [9].

Theoretically, en bloc resection of bladder tumor (ERBT) could reduce recurrence rates due to complete tumor removal and reduction of tumor dispersal [9, 10]. For this reason, ERBT was recently identified as a promising TUR technique at the meeting of the European Association of Urology (EAU) section of Uro-Technology and the section of Uro-Oncology [11].

Innovative resectoscope modifications or alternative instruments have been developed to achieve the intention of en bloc resection. Initially, en bloc resection was described by Ukai et al. [12] which allows en bloc resections by making a circular incision around the tumor and including a 0.5-cm safety margin with a J-shaped needle electrode. Recently, Hurle et al. [13] also report a series of prospective data using the same J-shaped needle electrode.

Matthew described an endoscopic snare resection technique (ESRBT) using an electrosurgical polypectomy snare to achieve en bloc resection [9]. A relatively new idea was the grasp and bite technique, which can effectively be applied to small, flat, sessile lesions, but may not be suitable for a larger tumor [14]. Laser is another efficient alternative energy source to achieve en bloc resection of the bladder tumor [2, 15]. In addition to modified electrical loops and laser systems, water-jet-induced enucleation was reported by Nagele et al. [16] and Fritsche et al. [17] proving feasibility en bloc resection in tumor sizes up to 7.5 cm.

In this study, we reported yet another feasible and safe en bloc resection technique using only a conventional resectoscope and loop without any accessory equipment. The safety and efficacy of the novel technique were comparable to conventional TURBT. Each “small bite” of RERBT was performed under direct vision, which reduced the risk of cutting the bladder wall too deep or causing perforation. Once normal glistening yellow fat is seen between the muscle layers [6], the depth of incision was secured. Usually, it takes only one or two “small bites” to reach the muscle layer.

In order to avoid charring the surrounding mucosa and bladder perforation, the movement of the electrode is somewhat fast in conventional TURBT, resulting in difficult depth control of resection [18]. However, using the “small bite” cutting technique, the resection was performed step by step without a hasty movement and was safer than the conventional resection.

The reported risk of obturator nerve stimulation during TURBT is from 10.6 to 11.0%, which is the major reason for bladder perforation [14]. Comparable to the C-TURBT group, obturator reflex could not be avoided in the RERBT group (22.5 vs 24%, *P* = 0.867). However, by using general anesthesia and a muscle relaxant, obturator reflex could be reduced to an extent that it seldom caused severe complication such as perforation due to gentle “small bite” cutting fashion. Only two cases of bladder perforation occurred in the first 15 series. Both patients were discharged after 6 days of catheterization without any complication.

There is increasing evidence which proves that ERBT improves the quality of the resected specimens, and some results indicate that residual tumor in the second resection may be found in lower percentages [13, 19, 20]. The presence of muscular propria in the tumor specimen is crucial for accurately discriminating between stages pT1 and pT2 [21, 22]. However, conventional TURBT inevitably leads to fragmented tumor specimen with poor anatomic orientation due to piecemeal resection fashion. This will cause a substantial risk of understaging mainly for patients whose TURBT specimens do not contain the muscular propria [23, 24]. In additional, the existence of lamina propria in the RERBT specimen without obvious charring could facilitate accurate pathological diagnosis.

The muscle positive rate of tumor base biopsy in the RERBT group was 100%, while that of the C-TURBT group was only 70% (*P* < 0.01). Moreover, only 54% of resected specimen in the C-TURBT group included the muscularis propria, compared to 100% in the RERBT group (*P* < 0.01). The lower muscle positive rate both in the resected specimen and the tumor base biopsy in C-TURBT was attributed to incomplete resection. Tissue charring or vaporization of the tumor itself may cause difficulties for pathologists in identifying muscle layers. On the contrary, our approach used a technique involving dissection primarily into normal-appearing tissues under direct vision and avoided excessive burning of tissues, reducing the possibility of pathological false judgment of specimen postoperatively.

With the “small bite” resection fashion, the operative time of RERBT was estimated to be longer. Actually, time consumption of the RERBT group was comparable to the C-TURBT group, (36 ± 11.8 min vs. 34 ± 13.6 min, *P* = 0.464). This may be due to the precise and efficient incision of RERBT under a clearer vision. On the contrary, poor visibility secondary to intraoperative bleeding might increase the difficulty of the operation and lead to residual tumors on the C-TURBT arm [25].

Similar to conventional C-TURBT, the RERBT technique could have difficulties in a posterior- or dome-located tumor due to inconvenient resection angle. Although this could be overcome by using suprapubic pressure and nearly emptying the bladder, we do not recommend performing the RERBT procedure in dome-located tumors.

It is possible to retrieve tumors up to 4.5 cm by using a nephroscopy sheet and a laparoscopic grasp [26]. However, the loop might be covered by the tumor itself during the retrograde resection when it was more than 4.0 cm in diameter. Therefore, the protruding tumor can be first removed superficially by the conventional TURBT followed by the novel technique to remove the remaining stalk and base lesion, avoiding disruption of the tumor base architecture. Furthermore, since one of the advantages of RERBT is to provide a better quality of resection of the tumor base, we believed that it would be more suitable for a larger tumor (>2 cm). Our study had certain inherent limitations due to its retrospective nature. First, the two techniques were performed by two individual surgeons, leading to a potential selection bias. Second, considering this small population and insufficient follow-up time, we were unable to conclude the advantage of the novel technique in terms of the recurrent-free survival. Further prospective study with a larger population and long-term follow-up is warranted.

## Conclusions

We report a novel procedure of en bloc resection of non-muscle-invasive bladder tumors with conventional monopolar resection setting. Compared to conventional TURBT, this technique is safe and effective with the added merits of obtaining better quality tumor specimens which may allow for accurate histopathological diagnosis and staging.

## Abbreviations

C-TURBT: Conventional transurethral resection of bladder tumor
EAU: European Association of Urology
ERBT: En bloc resection of bladder tumor
ESRBT: Endoscopic snare resection technique
NMIBC: Non-muscle-invasive bladder cancer
RERBT: Retrograde en bloc resection of bladder tumor
TURBT: Transurethral resection of bladder tumor
WHO: World Health Organization
